# Pathways from Mindfulness to Career Adaptability: Emotional Intelligence and Psychological Capital as Mediators

**DOI:** 10.3390/ejihpe16050063

**Published:** 2026-04-30

**Authors:** Getachew Tassew Woreta, Girum Tareke Zewude

**Affiliations:** Department of Psychology, Wollo University, Dessie 1145, Ethiopia

**Keywords:** mindfulness, emotional intelligence, psychological capital, career adaptability, PLS-SEM, mediation, university students

## Abstract

Background: In an era characterized by rapid technological disruption and vocational uncertainty, Career Adaptability (CA) has emerged as a critical meta-competency for university students transitioning into the workforce. While the importance of CA is well-documented, the internal mechanisms that foster it remain under-explored. This research adopts a resource-based perspective to investigate how Mindfulness—a state of non-judgmental present-moment awareness—acts as a catalyst for career readiness. Specifically, this study examines a dual-mediation model, proposing that Mindfulness enhances Emotional Intelligence (EI) and Psychological Capital (PsyCap) (comprising hope, efficacy, resilience, and optimism), which in turn bolsters an individual’s capacity to adapt to changing career landscapes. By integrating these four constructs, the study provides a comprehensive framework for understanding how “being present” (Mindfulness) translates into “being prepared” (Career Adaptability) through the cultivation of emotional and psychological resources. Methods: The study collected data from 705 final-year students at Wollo University (male = 399 and female = 306). The study employed several well-established instruments: the Compound Psychological Capital Scale (CPC), the Five Facet Mindfulness Questionnaire (FFMQ), the Wong and Law Emotional Intelligence Scale (WLIES), and the Career Adapt-Abilities Scale (CAAS). These instruments were rigorously evaluated for their psychometric applicability within the Ethiopian context. Results: PLS-SEM analysis revealed: (a) direct and positive influences of mindfulness, PsyCap, and EI on career adaptability; (b) partial and positive mediation effects of PsyCap and EI in the mindfulness-career adaptability link; (c) a serial mediation effect of mindfulness through PsyCap and EI; and (d) the proposed model explained a substantial amount of variance in university students’ career adaptability. Conclusions: Despite its strengths, the study acknowledged certain limitations and discussed potential implications for enhancing career adaptability, highlighting the benefits of cultivating mindfulness.

## 1. Introduction

The rapid advancement of technology together with globalization brings about widespread automation and digitization, often presenting individuals with situations that are unfamiliar, unpredictable, and demanding, and eventually contributing to employment insecurity (Biemann et al., 2012; Parmentier et al., 2019). These changes require people to have a wider range of capabilities and psychosocial resources to successfully navigate through them. In this regard, young people are highly in demand because they encounter numerous transitions and challenges that can profoundly determine their immediate and long-term educational and career trajectories. For instance, the ever-changing world of work often presents new university graduates with multiple challenges (e.g., unemployment, high competitiveness, uncertainty of the current and future economic contexts), leading to increasing instability and uncertainty in managing their careers. The challenging and stressful nature of the transition from university to work life is expected and understood, but it has become more fear-provoking recently due to rapid technological advancements, increasing digitalization, high unemployment rates, and the volatile labor market. Unemployment of graduates is a prevailing worldwide issue, though it remains an ever-increasing problem in Ethiopia. Recent evidence, for example, indicates a 61.7% unemployment rate in rural Ethiopia (Olkeba et al., 2023). According to Niken et al. (2023)**,** Ethiopia’s economy has consistently experienced high inflation and unemployment. The pressure of high-level inflation in the country typically continues to challenge the job creation process, which in turn dramatically and consistently increases unemployment among new graduates. Such context suggests that present-time graduates need to have more adaptability and resilience to cope with the uncertainty they face (de Abreu et al., 2024). The capability to adapt to changes and uncertainties is becoming more pertinent in this ever-dynamic world of work than before for career development and success (Bocciardi et al., 2017; Santisi et al., 2018). With this understanding, the need to develop one’s ability to adapt to the changing world as an essential prerequisite for success is underscored (Chen et al., 2020).

Recently, there is an understanding that “individuals must continuously adapt throughout the life course to respond effectively to changing personal needs and environmental demands and opportunities to remain productive, purposeful, and gainfully employed” is one of the most important psychosocial resources that enables individuals to navigate changes in career roles and to be successful in their career development and other aspects of life (Chen et al., 2020). With this recognition, career scholars and practitioners worldwide agree that career adaptability is a fundamental construct for understanding how people behave in their jobs and formulating interventions to help them change themselves and their situations so that they get better at navigating work environments, increase their chances of employability, and develop the self-controlling thoughts, feelings, actions, and attitudes that are necessary for career success and satisfaction (Salovey & Mayer, 1990). Overall, career adaptability has been seen as a meta-competency that enables one to make changes in self and situations needed for managing transitions, tasks, and traumas associated with career exploration, career choice, and work adjustment.

Career adaptability and resilience are essential personal resources that help individuals design their careers, better use their environment’s resources, and enhance their employability (Rossier et al., 2017). Evidence has shown that career adaptability is crucial for mastering the school-to-work transition (Konstam et al., 2015), increasing an individual’s chances of finding a suitable job (Koen et al., 2012), and career satisfaction (Ye et al., 2024). It has also been indicated that career adaptability enhances individuals’ sense of power and life satisfaction (Hirschi, 2009)**,** mitigates mental health problems (C. Xu et al., 2020), and enhances career satisfaction (Zacher, 2015). Recently, career adaptability has been considered a core element of life design and satisfaction (Baer et al., 2006). That is, because of the potential utility of career adaptability in enhancing positive outcomes in multiple areas of life, it has increasingly drawn researchers’ attention. Nevertheless, little is known about what factors contribute to career adaptability development; especially in the Ethiopian context, nothing has been started. To design evidence-based career interventions that increase individuals’ level of adaptation and ability to manage the changing situations, further efforts are needed to identify important predictors of career adaptability. So, the present study aimed to predict career adaptability using the model that includes mindfulness, emotional intelligence, and psychological capital, the latter two being the mediators between mindfulness and career adaptability. Achieving this purpose is highly substantial because career adaptability enhances multiple positive outcomes and it can be cultivated (Chen et al., 2020). The findings would make available valuable insights to practitioners regarding how to support individuals, particularly graduates, in developing career adaptability during the early career stage to navigate today’s dynamic world of work. 

### 1.1. Theoretical Foundations of the Study

Career adaptability—defined as the readiness and capacity to manage predictable and unpredictable career tasks and transitions through planning, exploration, and coping with uncertainty (Savickas, 2005), offers a useful lens for understanding how young adults navigate contemporary work environments. For Generation Z (Gen Z), who enter the workforce amid rapid technological change, skills mismatch, and frequent transitions from education to employment, adaptability is especially salient. The contemporary labor market is characterized by instability, heightened competition, and demands for continuous learning and cognitive flexibility. These pressures make the transition from education to employment increasingly complex and uncertain, posing particular challenges for Generation Z graduates (those born between 1995 and 2012). Although Gen Z is highly fluent with digital technologies, they still face significant obstacles to securing stable and meaningful work. This issue is especially pronounced in Ethiopia, where undergraduate university students are predominantly members of Gen Z and enter a labor market constrained by limited opportunities and intensifying competition. Within this context, it is critical that young people possess psychological resources that enable them to adapt effectively to career-related uncertainty and complexity (De Vos et al., 2020).

Empirical research identifies career adaptability as a psychological capability linking personal resources to positive outcomes such as achievement motivation, work engagement, and life satisfaction (Coetzee et al., 2017; Ginting, 2022; Rudolph et al., 2017). In the integrated framework of this dissertation, mindfulness is positioned as an upstream personal resource that may promote adaptive career responses by fostering present-centered awareness and emotional regulation—capacities that help individuals appraise career challenges more constructively and engage in proactive planning and exploration.

Career Construction Theory (CCT) and Positive Psychology provide a coherent foundation for understanding career adaptability as a malleable capability that helps individuals navigate work-related tasks, transitions, and traumas across the lifespan (Savickas & Porfeli, 2012; Seligman & Csikszentmihalyi, 2000; Coetzee et al., 2017). CCT emphasizes that individuals contribute actively to their own career development through adaptability, personal meaning, and narrative identity. A central claim in CCT is that effective career development is an ongoing adaptive process in which individuals adjust their needs to the prevailing demands of their context (Savickas et al., 2009; Savickas & Porfeli, 2012). Moreover, CCT proposes individual differences in adaptability resources that are essential for responding to changing career conditions. In this sense, people are not merely shaped by external factors; they also shape their futures through their choices and actions.

Within CCT, career adaptability is conceptualized as a psychosocial construct reflecting individuals’ resources for coping with current and anticipated tasks, transitions, and traumas in occupational roles (Savickas & Porfeli, 2012). Rottinghaus et al. (2005) further describe it as the perceived ability to adapt to and leverage future changes, handle new job responsibilities with comfort, and build resilience when unexpected disruptions occur. Accordingly, career adaptability represents the attitudes, abilities, and behaviors needed to manage career-related tasks and challenges (Zewude et al., 2025a). CCT proposes four dimensions—concern, control, curiosity, and confidence—which capture, respectively, looking ahead to envision the future, owning one’s life-career decisions, exploring work and career opportunities, and building confidence to solve problems (Salovey & Mayer, 1990). 

Career adaptability is important not only for employability but also for broader career success. Hecht and Allen (2009) argue that individuals with high adaptability can actively set goals, take intentional actions, and achieve psychological success, capacities that are vital for career development. When career adaptability has become a fundamental theoretical and empirical construct for navigating the increasingly complex world of work, CCT has emerged as a prominent meta-theoretical perspective for understanding vocational behavior dynamics across the lifespan (Savickas, 2005; Savickas et al., 2009; Savickas & Porfeli, 2012). Consistent with this view, Santilli et al. (2020) highlight how career adaptability supports the management of developmental career tasks, transitions, and challenges, positioning it as a valuable theoretical and empirical construct for understanding vocational behavior.

Positive Psychology complements CCT by focusing on strengths and psychological resources that enable individuals to flourish and remain resilient in the face of career challenges (Seligman, 2011). Mindfulness (Baer et al., 2006) is one key psychological resource within Positive Psychology, alongside constructs such as psychological capital (PsyCap; F. Luthans et al., 2007) and emotional intelligence (EI) (Pirsoul et al., 2023). Mindfulness—defined as an intentional and systematic present-moment experience conducted without judgment or analysis (Baer et al., 2006)—enhances self-awareness and emotional and cognitive awareness, which are essential in increasingly complex career contexts (Parmentier et al., 2019). PsyCap, a higher-order construct consisting of hope, efficacy, resilience, and optimism (F. Luthans et al., 2007, 2015), reflects a motivational state of mind that supports perseverance and confident goal striving while enabling individuals to overcome challenges. Therefore, PsyCap plausibly contributes to career adaptability by supporting goal-directed action and resilient coping. In combination with mindfulness, these resources create an explanatory pathway for how individuals sustain adaptive responses in career transitions.

Mechanistically, mindfulness may function as an upstream influence by cultivating moment-to-moment awareness and non-reactivity. These processes can increase cognitive clarity, reduce rumination, and heighten awareness of vocational interests and contextual demands. Mindfulness supports career adaptability by creating psychological space for deliberate processing, which strengthens planning and exploration behaviors central to adaptability (Hülsheger et al., 2013). In addition, mindfulness plausibly leads to higher EI because the attentional and emotion-regulation capacities strengthened through mindfulness overlap with core EI components such as emotion perception, emotion understanding, and emotion management (Salovey & Mayer, 1990). Empirical work in Gen Z samples suggests that EI positively predicts career adaptability and achievement-oriented outcomes. For example, Ginting (2022) found that EI positively predicted career adaptability and that adaptability partially mediated the relationship between EI and achievement motivation. In this model, EI is positioned as a proximal mediator linking mindfulness to career adaptability: mindfulness strengthens emotional competencies, which in turn help individuals interpret career feedback, manage setbacks, and sustain goal-directed career behaviors.

Psychological capital offers a parallel and additional mediating pathway from mindfulness to career adaptability. Mindfulness may enhance PsyCap by reducing rumination, increasing cognitive flexibility, and supporting adaptive coping. As a result, individuals may develop stronger efficacy beliefs, hopeful goal striving, optimistic appraisal, and greater resilience when facing setbacks (F. Luthans et al., 2007, 2015; Zewude et al., 2025a). PsyCap has been shown to predict well-being and adjustment outcomes in Gen Z samples (e.g., J. Xu & Choi, 2023), and intervention research indicates that PsyCap components are developable. This supports the plausibility of targeting PsyCap to improve career-related outcomes. Higher PsyCap provides Gen Z individuals with motivational and resilient resources to engage in career planning, exploration, and adaptive coping, core elements of career adaptability that promote retention and well-being. In addition, a systematic review by Nu’ma and Mangunsong (2024) reports mixed findings regarding Gen Z students’ Psychological Capital (PsyCap) in relation to job-market readiness: some studies indicate that Gen Z PsyCap is lower than that of previous generations, while others find it to be in the medium to high range. These differences suggest that, although concerns about lower PsyCap among Gen Z may exist, this group also shows meaningful potential to develop strong psychological resources when facing career-related challenges.

Taken together, the evidence supports a two-layer mediation model in which mindfulness promotes career adaptability through (a) enhanced EI, which strengthens emotion-related competencies critical for adaptive career decision-making, and (b) strengthened PsyCap, which supplies motivational and resilience resources for sustained goal pursuit. EI and PsyCap therefore function as proximal intrapersonal skill sets and psychological resources through which mindful awareness translates into concrete career-adaptive behaviors. Career adaptability itself—reflected in concern, control, curiosity, and confidence—then enables adaptive behaviors (planning, exploration, and coping), which subsequently contribute to improved achievement motivation, work engagement, employability, and life satisfaction (Buyukgoze-Kavas, 2016; Hecht & Allen, 2009; Savickas, 1997). 

This integrative framework is also cohort-sensitive. Career resources and adaptability may differ across groups (Coetzee et al., 2017), indicating the importance of examining heterogeneity within Gen Z (e.g., students versus early-career employees and sectoral differences). Practically, because EI and PsyCap are amenable to training and intervention, mindfulness-based programs that explicitly target emotional competencies and PsyCap components may provide viable routes for improving career adaptability among Gen Z. This could strengthen engagement, retention, and life satisfaction in volatile labor markets (F. Luthans et al., 2007, 2015; Bocciardi et al., 2017). 

In sum, integrating CCT and Positive Psychology yields a parsimonious, actionable model: upstream mindful awareness enhances intrapersonal competencies (EI) and psychological resources (PsyCap), which together build career adaptability. Career adaptability then supports adaptive career behaviors that foster achievement, engagement, and life satisfaction, outcomes particularly important for Generation Z navigating contemporary labor markets (Savickas & Porfeli, 2012; Seligman & Csikszentmihalyi, 2000; Coetzee et al., 2017).

### 1.2. Association Among the Study Constructs 

#### 1.2.1. Mindfulness and Career Adaptability

Being actively aware and engaged in the present moment without judgment defines the construct of mindfulness (Kabat-Zinn, 2003). Conscious and nonjudgmental attention to the present moment is the fundamental feature of mindfulness. Most contemporary conceptualizations of mindfulness consider adopting an attitude of openness or acceptance toward one’s experience as a critical element of the construct (Creswell, 2017). With this conceptualization, mindfulness is assumed to foster psychological flexibility and adaptability, thereby enhancing an individual’s ability to respond adaptively to career challenges. Mindfulness is related to low levels of stress and high psychological well-being (Creswell, 2017; Palmer & Rodger, 2009). Mindfulness, characterized by curiosity, openness, and acceptance (Bishop et al., 2004), allows individuals to make informed decisions and adapt their thinking to work environment challenges and changes. Evidence indicates that mindfulness reduces stress, builds resilience, improves problem-solving and decision-making abilities, and leads to greater career satisfaction (Jacobs & Blustein, 2010; Kiken et al., 2017), suggesting the possibility of its direct link to career adaptability. According to de Abreu et al. (2024, p. 432), “Although no empirical research has been done on the association between mindfulness and career adaptability, there seem to be conceptual linkages between the two concepts”, contending that mindfulness positively influences career adaptability (Zewude et al., 2025b). However, their study did not find supportive empirical evidence for what they assumed. The present study’s theoretical consideration is that mindfulness would enhance career adaptability by (a) promoting awareness of changes, enabling individuals to be future-oriented and more proactive about future tasks and challenges in their careers (concern), (b) fostering purposeful behavior, encouraging responsibility in considering alternatives and making career-related decisions (control), (c) encouraging exploration of the potential future selves and opportunities (curiosity), and (d) boosting beliefs in ability to convert goals into reality and overcome obstacles (confidence). Therefore, it was hypothesized that mindfulness would directly predict career adaptability. 

#### 1.2.2. Linking Mindfulness to Career Adaptability Through Emotional Intelligence

To fully comprehend the impact of mindfulness on career adaptability, it is essential to delineate the underlying psychological mechanisms driving this relationship. Mindfulness likely fosters career adaptability through multiple interconnected pathways, with the cultivation of emotional intelligence (EI) emerging as a particularly salient mechanism. Emotional intelligence is defined as “the ability to monitor one’s own and others’ feelings and emotions, to discriminate among them, and to use this information to guide one’s thinking and actions” (Hirschi et al., 2015). This conceptualization posits that EI encompasses the critical skills required to recognize, process, and regulate emotions. Consequently, these competencies profoundly influence an individual’s behavioral self-regulation, adeptness in navigating complex social dynamics, and capacity for optimal decision-making (Ye et al., 2024).

The instrumental role of EI is well-documented across various vocational domains, yielding empirical support for its positive impact on career decision-making, career commitment (Brown et al., 2003), career success (García & Costa, 2014), and overall employability (Hodzic et al., 2015). Beyond vocational outcomes, EI is associated with broader life adaptability, mitigated suicide risk (Domínguez-García & Fernández-Berrocal, 2018), enhanced academic performance (Lyons & Schneider, 2005), and enriched interpersonal relationships (Schutte et al., 2001). However, the present study focuses specifically on the intersection of EI and career adaptability. To successfully navigate various life domains and career transitions, both EI and career adaptability function as vital psychosocial resources (Coetzee & Harry, 2014; Parmentier et al., 2019).

While the directional precedence between the two has historically been debated, a robust and growing body of empirical evidence positions EI as a critical antecedent to adaptability. For instance, Pong and Leung (2023) demonstrated that all facets of EI are significantly associated with heightened career adaptability among Chinese youth. Longitudinal evidence further reinforces this predictive relationship (Parmentier et al., 2019). Parmentier et al. (2019) established that baseline EI (Time 1) significantly predicts subsequent career adaptability (Time 2), even after controlling for socio-demographic factors and prior adaptability levels. Similarly, Zewude et al. (2025a) revealed that trait EI positively influences career adaptability—and subsequently reduces career indecision—independent of cognitive intelligence, biological sex, and personality traits. The capacity to manage one’s emotions directly bolsters all four dimensions of career adaptability, and a positive emotional disposition has been longitudinally linked to enhanced adaptive capacities. A comprehensive meta-analysis by Pirsoul et al. (2023) further solidifies this robust association. Theoretically, this implies that individuals possessing high EI are equipped with accurate emotional perception and superior regulatory capabilities, enabling them to execute highly adaptive responses to vocational challenges.

Crucially, existing literature provides substantial evidence for EI’s role as a mediating mechanism between mindfulness and various positive psychological outcomes. Mediation studies have demonstrated that EI bridges the gap between mindfulness and reduced burnout (Y. Wang et al., 2022), subjective well-being (Schutte & Malouff, 2011), positive psychological traits (e.g., gratitude, empathy, resilience) (Schutte & Meynadier, 2024), and emotional exhaustion (Xie et al., 2021). Specific components of EI, such as emotion regulation and utilization, have also been shown to mediate the mindfulness-stress relationship (Bao et al., 2015). This aligns with foundational findings that mindfulness practices actively enhance EI (Jiménez-Picón et al., 2021; Zhou et al., 2022), suggesting that greater mindfulness naturally facilitates higher emotional intelligence.

Building on prior theoretical and empirical foundation—specifically, the established links between mindfulness and EI, and between EI and career adaptability—the present study posits a predictive mediational model. We hypothesize that the positive influence of mindfulness on career adaptability is not merely direct, but is significantly transmitted through the cultivation and application of emotional intelligence.

#### 1.2.3. Linking Mindfulness to Career Adaptability Through Psychological Capital 

Psychological Capital (PsyCap) refers to an individual’s positive psychological state of development and is typically conceptualized as comprising four capacities (F. Luthans et al., 2007, 2015): (1) self-efficacy, or confidence in one’s ability to mobilize motivation and effort to succeed in challenging tasks; (2) optimism, defined as a generalized tendency to hold positive expectations about present and future outcomes; (3) hope, reflected in perseverance toward goals alongside the ability to generate alternative routes when obstacles arise; and (4) resilience, the capacity to recover from setbacks, adapt to disruption, and potentially grow through adversity. PsyCap is increasingly recognized as a developable psychological resource rather than a fixed trait, meaning it can be strengthened over time through supportive experiences and targeted interventions (B. C. Luthans et al., 2014). Consequently, clarifying the factors that foster PsyCap, as well as establishing its predictive power for outcomes such as career adaptability, is an important research priority.

Mindfulness and PsyCap are both regarded as valuable personal resources that support effective functioning across life domains, including education and work. Mindfulness, commonly understood as present-moment awareness coupled with a nonjudgmental and accepting stance toward one’s experiences, may help individuals regulate attention and emotions, reduce automatic reactivity, and respond more flexibly to stressors. PsyCap, in turn, reflects a cluster of motivational and cognitive strengths that sustain confidence, goal pursuit, positive expectancy, and adaptive coping. Although both constructs appear highly relevant to career development, the mechanism through which mindfulness may translate into greater career adaptability—particularly through the enhancement of PsyCap—has not been sufficiently examined. Addressing this gap is important because career adaptability depends not only on skills and opportunities, but also on psychological resources that enable individuals to anticipate change, cope with uncertainty, and persist through transitions (Zewude et al., 2025b).

Empirical research provides growing support for the notion that PsyCap helps explain how mindfulness contributes to a variety of positive outcomes. Prior studies have shown that PsyCap mediates the relationship between mindfulness and study engagement (Miralles-Armenteros et al., 2021), English language anxiety (Gordani & Sadeghzadeh, 2023), creativity (He, 2024), and mental well-being (Roche et al., 2014). Using a three-wave time-lagged design, Li et al. (2023) further demonstrated that PsyCap mediates the mindfulness–creativity association over time, lending additional credibility to a pathway in which mindfulness precedes the development of PsyCap and subsequently predicts beneficial outcomes. Evidence from intervention research also points in the same direction. For example, Zhou et al. (2022) reported that mindfulness training can enhance PsyCap, suggesting that mindfulness is not merely correlated with PsyCap but may play an active role in building it.

Theoretically, several mechanisms may explain why mindfulness could foster PsyCap. By strengthening attentional control and reducing distractions, mindfulness may facilitate mastery experiences and persistent effort, thereby supporting self-efficacy. By encouraging more balanced appraisals and reducing rumination, mindfulness may contribute to optimism, helping individuals interpret setbacks as temporary and manageable rather than as definitive failures. Mindfulness can also strengthen hope by helping individuals remain anchored to valued goals while flexibly adjusting strategies when obstacles emerge, a process consistent with pathways thinking. Finally, mindfulness may enhance resilience by improving emotion regulation and stress recovery, enabling individuals to respond to adversity with greater composure and adaptability.

At the same time, the literature indicates that the relationship may not be exclusively one-way. An exception has been noted in which mindfulness significantly mediates the association between PsyCap and burnout, implying that individuals with higher PsyCap may become more mindful over time (Liu & Du, 2024). This finding raises the possibility of reciprocal effects, where mindfulness and PsyCap mutually reinforce one another in certain contexts. Nevertheless, the broader pattern of evidence—particularly time-lagged and training-based findings—supports the proposition that mindfulness can contribute to the development of PsyCap. Accordingly, it is reasonable to hypothesize that mindfulness positively influences psychological capital.

Research directly linking PsyCap to career adaptability is relatively limited, but the available findings are consistent and theoretically coherent. PsyCap has been found to relate directly to career adaptability (Coetzee et al., 2023), suggesting that individuals with stronger psychological resources are better prepared to handle career-related tasks, transitions, and uncertainties. Studies also indicate that key components of PsyCap are relevant. Hope, for instance, has been associated with career adaptability (Lin, 2024), aligning with the idea that adaptive career behavior requires sustained goal pursuit and the ability to generate alternative pathways. Similarly, resilience, hope, and optimism have been identified as potential predictors of career adaptability(Buyukgoze-Kavas, 2016; Safavi & Bouzari, 2019). The authors underscore the importance of recovering from setbacks, maintaining motivation, and expecting that effort can yield positive outcomes in dynamic career environments. Additional evidence suggests that PsyCap may influence career engagement indirectly through career adaptability, implying that psychological resources can first enhance adaptive readiness and coping capacity, which in turn promote proactive career behaviors such as planning, exploration, networking, and skill development (Zyberaj et al., 2022).

Taken together, these findings support a mediational explanation in which mindfulness contributes to higher PsyCap, and PsyCap, in turn, facilitates career adaptability. Mindfulness may help individuals remain psychologically present and flexible, thereby strengthening the confidence, hopeful goal pursuit, optimistic outlook, and resilient coping patterns that constitute PsyCap. These PsyCap capacities then equip individuals to manage career disruptions, respond constructively to new demands, and persist through uncertainty. Therefore, building on the evidence that PsyCap can be shaped by mindfulness and that PsyCap is associated with career adaptability, PsyCap is proposed to mediate the relationship between mindfulness and career adaptability.

### 1.3. Empirical Studies in Africa

Studies in Africa also indicate that emotional intelligence (EI) and career adaptability are crucial psychosocial meta-capacities for successful adaptation across life domains, including careers (Coetzee & Harry, 2014). However, little is known about how EI relates to Savickas (2005), career adaptability. In response, a cross-sectional survey of 409 early-career Black call center agents (mean age = 32) in three outsourced financial call centers in Africa used canonical correlation analysis and structural equation modeling, confirming the predictive validity of EI for career adaptability (Coetzee & Harry, 2014). The results showed that managing one’s own emotions contributed most to explaining overall EI and to the variance in overall career adaptability across its four domains—career concern, career control, career confidence, and career curiosity—highlighting the importance of developing EI to strengthen career adaptability (Coetzee & Harry, 2014). 

Beyond EI, emerging African evidence links psychological capital (PsyCap) to key work and student outcomes. For example, Sifuna Mayende et al. (2026) found that PsyCap directly influences both job satisfaction and work engagement, with job satisfaction mediating the relationship between PsyCap and work engagement, suggesting practical value for management and policymakers to enhance PsyCap through strategies such as flexible work programmes (Sifuna Mayende et al., 2026). Similarly, (Baluku et al., 2021) examined Ugandan and Kenyan final-semester university students (N = 516) and found substantial positive direct effects of PsyCap on perceived employability, readiness for school-to-work transitions (STWT), and career satisfaction. Their double-mediation results further indicated that PsyCap affects STWT readiness indirectly through career engagement and internal perceived employability, and that PsyCap affects career satisfaction indirectly via career engagement and external perceived employability (Baluku et al., 2021). Other studies extend these findings by synthesizing and integrating EI and PsyCap evidence. Tjimuku et al. (2025), in a review that screened 255 articles and included 57 studies, systematically synthesized conceptualizations, models of EI and PsyCap, and empirical findings, reporting a generally positive association between EI and PsyCap. The review further indicated that this EI–PsyCap association relates to outcomes such as job performance and employee psychological well-being, while noting limitations including potential publication bias and reliance on existing literature (Tjimuku et al., 2025). Alutaya and Guhao (2023) likewise reported significant correlations among PsyCap, academic job satisfaction, EI, and work engagement, and identified an overall best-fit model in which PsyCap, academic job satisfaction, and EI directly predicted work engagement. In their model, PsyCap was represented primarily by hope and optimism; academic job satisfaction by “my work itself” and interpersonal relationships; EI by empathy and motivating oneself; and work engagement by absorption and dedication, suggesting implications for teacher advancement programmes and educational organizational initiatives (Alutaya & Guhao, 2023).

Within public-sector settings, Coetzee et al. (2023) found positive associations among study variables for Black African employees (N = 412; mean age = 38.79). Mediation analyses showed that technological adaptivity, agile learning, optimism, and hope activated career concern, career control, and career curiosity, which in turn supported self-reliance, personal resilience, and work resilience. The study also found direct effects of career agility and PsyCap on career adaptedness modes, including career resilience and career satisfaction, thereby enriching career adaptation theory and supporting organizational career development practice (Coetzee et al., 2023). Finally, (Zewude et al., 2025b) in Ethiopia (N = 1026 undergraduate students from three public universities) showed, through a parallel mediation model, that career adaptability (CA) and career decision-making self-efficacy (CSE) positively predicted students’ basic psychological needs satisfaction (BPNS). BPNS and mindfulness also had substantial positive direct effects on students’ career choice, while CA and CSE indirectly predicted future career choice through BPNS and mindfulness. These results further indicated that BPNS and mindfulness mediated (fully and partially) the relationships among CA, CSE, and career choice, emphasizing the value of strengthening both constructs to support informed career decisions and personal/professional growth (Zewude et al., 2025b). 

Overall, the reviewed evidence indicates that psychological capital (PsyCap) and emotional intelligence (EI) are significant predictors of career adaptability, with effects commonly observed both directly and indirectly through relevant mediating mechanisms such as job/work or career-related psychological states and related outcomes.

### 1.4. The Present Study

The primary goal of this research is to predict university students’ career adaptability through a comprehensive structural model that integrates mindfulness (Baer et al., 2006), emotional intelligence (EI; Salovey & Mayer, 1990), and psychological capital (PsyCap; F. Luthans et al., 2007, 2015), specifically positing the latter two as partial mediators of the pathways from mindfulness to career adaptability (Savickas, 2005; Savickas et al., 2009). This study is driven by the overarching objective of redefining career adaptability as a holistic, psychologically driven capacity rather than a mere behavioral skill set. To achieve this, the study is structured around several broad objectives: 

First, the study aims to achieve a sophisticated theoretical synthesis by bridging Career Construction Theory (CCT, Savickas, 2005) and Positive Psychology (Seligman, 2011). While CCT provides a robust explanation of how individuals navigate vocational transitions, it often overlooks the “internal engine” or the specific psychological strengths that fuel this process. By framing career adaptability as a product of positive psychological resources, this research moves beyond a purely behavioral view of career development to explore how an individual’s internal psychological flourishing drives their ability to construct a career in a volatile market. 

Second, this research seeks to expand the empirical landscape of career development by moving beyond the “thin slice” of existing scholarly work. Current literature is largely limited to the effects of career adaptability or its relationship with stable demographic factors—such as gender and age (Hirschi, 2009; Rottinghaus et al., 2005)—and personality traits (Guan et al., 2017; Hirschi, 2009; Jiang, 2017; Rudolph et al., 2017). This study addresses this limitation by examining a single comprehensive model that simultaneously tests the structural paths among mindfulness, EI, and PsyCap, providing a more granular and nuanced explanation of the predictors of adaptability that is currently absent from the broader literature. 

Third, the study is designed to address the critical need for contextual diversity and inclusivity in psychological research. Despite the documented impact of culture on career adaptability (Chen et al., 2020), there is a near-total absence of research conducted on the Ethiopian population. By focusing on Ethiopian higher education—a landscape characterized by rapid transition and economic pressure—this study aims to uncover context-specific insights, address locally significant challenges, and ensure that the global body of knowledge in vocational psychology is representative of diverse perspectives (Zewude et al., 2025b). 

Fourth, a central objective of this research is to open the internal “black box” of the mindfulness-adaptability relationship. Building on the foundational work of Zewude et al. (2025a), this study shifts the focus from “need satisfaction” to “resource building”. It proposes that mindfulness does not operate in a vacuum; rather, it facilitates a heightened state of self-awareness that allows for the development of the “psychological infrastructure”—specifically, Emotional Intelligence and Psychological Capital (Hope, Efficacy, Resilience, and Optimism)—necessary for students to regulate career-related anxieties and persist through uncertainty.

Fifth, the study pursues a practical and interventionist goal by focusing on malleable psychological constructs. Unlike stable personality traits, mindfulness, EI, and PsyCap can be enhanced through training, counseling, and coaching (Chang et al., 2018; Di Fabio et al., 2013; Johnston et al., 2013; B. C. Luthans et al., 2014; Savickas & Porfeli, 2012). Consequently, the results of this study are intended to provide a practical roadmap for educators, career counselors, and policymakers to design and implement interventions that boost the career readiness of graduates, ensuring they are equipped to adapt to the demands of the modern, dynamic world. 

Sixth, and finally, this research has the indirect intention of contributing to the methodological advancement of the field within the region. By validating and localizing the measures of the constructs included in the study, the research ensures that the tools used to assess mindfulness, EI, PsyCap, and career adaptability are culturally and contextually relevant, thereby providing a reliable foundation for future research and practice in the Ethiopian context. Drawing upon the most recent scientific literature and the proposed theoretical framework, this study posits the following research hypotheses:

**Research** **Hypothesis** **1** **(RH1).** *There are significant positive interrelationships among mindfulness, emotional intelligence, psychological capital, and career adaptability.*

**Research** **Hypothesis** **2** **(RH2).** *Mindfulness exerts a significant, direct positive effect on (a) emotional intelligence, (b) psychological capital, and (c) career adaptability.*

**Research** **Hypothesis** **3** **(RH3).** *Emotional intelligence and psychological capital act as significant mediators in the relationship between mindfulness and career adaptability using Smart PLS 4.1.1.6.*

## 2. Methods

### 2.1. Research Design

The current study employed a quantitative research design with an associational approach, deemed well-suited to achieve the stated objectives.

### 2.2. Sample and Sampling

The participants were 705 randomly selected final-year students at Wollo University (56.6% male), Ethiopia. Initially, 728 questionnaires were collected, but the screening process excluded 23 cases either due to incompleteness or significant missing data. Consequently, the final sample consisted of 705 students (mean age = 22.61 years, SD = 0.91, range from 21 to 25 years). Being university students increases the diversity of the participants in various dimensions, making the findings more likely to be generalized. Final-year or graduating students were in focus because job hunting and adapting to uncertainties are the most immediate and relevant concerns for them, and thus, they can provide useful data on their ability to effectively prepare for and navigate career-related changes, challenges, and opportunities. Furthermore, the data gathered during this phase could serve as a baseline for longitudinal research if there is a need to examine the effects of career adaptability on long-term career success and satisfaction. Overall, final-year students are assumed to be a highly relevant and insightful population for research on career adaptability from multiple perspectives.

### 2.3. Measures

In the contextualization process for all measures used in the present study, an individual who was a native Amharic speaker and highly fluent in English first translated the instrument into Amharic. Next, two independent professionals back-translated the instrument into English. A third person then compared the back-translated versions, and the researcher thoroughly assessed each item for clarity of language and accuracy of meaning.

***Psychological Capital.*** This scale was assessed using the 12-item Compound Psychological Capital Scale (CPC-12R; F. Luthans et al., 2007, 2015; Lorenz et al., 2022; Timo et al., 2016). The scale uses response options ranging from 1 (strongly disagree) to 6 (strongly agree). It comprises four dimensions—hope, optimism, self-efficacy, and resilience—with three items measuring each dimension. Sample items include: “I am looking forward to the life ahead of me” (optimism), “I can solve most problems if I invest the necessary effort” (self-efficacy), “I can think of many ways to reach my current goals” (hope), and “I consider myself able to endure a great deal; I am not easily discouraged by failure” (resilience).

The scale has been validated across different contexts and has demonstrated good psychometric properties (Ikeda et al., 2023; Lorenz et al., 2022; Prochazka et al., 2025; Timo et al., 2016). In the present study, the reliability and convergent validity indices for the four dimensions were satisfactory: self-efficacy (α = 0.929, CR = 0.955, AVE = 0.876), hope (α = 0.886, CR = 0.930, AVE = 0.816), optimism (α = 0.869, CR = 0.920, AVE = 0.793), and resilience (α = 0.826, CR = 0.896, AVE = 0.742). All values exceeded the recommended thresholds of 0.70 for α and CR, and 0.50 for AVE. Discriminant validity was also supported, as the HTMT values ranged from 0.627 to 0.773, and the correlations between constructs were lower than the square root of each construct’s AVE.

***Mindfulness.*** Mindfulness was assessed using the 18-item Five Facet Mindfulness Questionnaire (FFMQ-18; Medvedev et al., 2018). Participants responded on a 5-point rating scale ranging from 1 (never or very rarely true) to 5 (very often or always true). The instrument consists of five subscales: Acting with Awareness (3 items; e.g., “I find it difficult to stay focused on what’s happening in the present moment”), Describe (5 items; e.g., “I’m good at finding words to describe my feelings”), Non-judging (3 items; e.g., “I make judgments about whether my thoughts are good or bad”), Non-reactivity (4 items; e.g., “I watch my feelings without getting carried away by them”), and Observing (3 items; e.g., “I pay attention to physical experiences, such as the wind in my hair or sun on my face”). Negatively worded items were reverse-coded so that higher scores indicated greater mindfulness. The FFMQ is one of the most widely used measures of mindfulness and has demonstrated adequate reliability and validity across diverse populations in both its original and short versions. In the present study, the reliability and convergent validity indices for the five dimensions were satisfactory: describe (α = 0.874, CR = 0.908, AVE = 0.665), Acting with awareness (α = 0.816, CR = 0.891, AVE = 0.732), Observe (α = 0.818, CR = 0.892, AVE = 0.733), Non-react (α = 0.831, CR = 0.888, AVE = 0.664), and Non-judge (α = 0.882, CR = 0.928, AVE = 0.811). All values exceeded the recommended thresholds of 0.70 for α and CR, and 0.50 for AVE. Discriminant validity was also supported, as the HTMT values ranged from 0.421 to 0.787, and the correlations between constructs were lower than the square root of each construct’s AVE.

***Emotional Intelligence.*** Emotional intelligence was measured using the 16-item Wong and Law Emotional Intelligence Scale (WLEIS; Wong & Law, 2002; Zewude et al., 2024a). The scale consists of four subscales, each represented by four positively worded items: Self-emotion appraisal, Others’ emotion appraisal, Regulation of emotion, and Use of emotion. Sample items include: “I have a good understanding of my own emotions” (Self-emotion appraisal), “I always know my friends’ emotions from their behavior” (Others’ emotion appraisal), “I have good control of my own emotions” (Regulation of emotion), and “I am a self-motivated person” (Use of emotion). Responses were recorded on a 5-point Likert scale ranging from 1 (strongly disagree) to 5 (strongly agree), with higher scores indicating greater emotional intelligence.

The WLEIS is widely used and has demonstrated adequate reliability, validity, and support for its four-factor structure across different contexts structure (Di et al., 2020; Lapalme et al., 2016; Zewude et al., 2024b). In the present study, the reliability and convergent validity indices for the four dimensions were satisfactory: others’ emotion appraisal (α = 0.873, CR = 0.913, AVE = 0.726), regulation of emotion (α = 0.830, CR = 0.887, AVE = 0.662), self-emotion appraisal (α = 0.853, CR = 0.901, AVE = 0.695), and use of emotion (α = 0.819, CR = 0.880, AVE = 0.648). All values exceeded the recommended thresholds of 0.70 for α and CR, and 0.50 for AVE. Discriminant validity was also supported, as the HTMT values ranged from 0.422 to 0.834, and the correlations between constructs were lower than the square root of each construct’s AVE.

***Career adaptability.*** Career adaptability was measured using the 24-item Career Adapt-Abilities Scale (CAAS; Savickas & Porfeli, 2012). Participants rated each item on a five-point Likert-type scale ranging from 1 = not strong to 5 = strongest. The scale comprises four dimensions—concern, control, curiosity, and confidence—with six items allocated to each dimension. Sample items include: “Thinking about what my future will be like” (concern), “Taking responsibility for actions” (control), “Becoming curious about new opportunities” (curiosity), and “Performing tasks efficiently” (confidence). Previous studies have reported acceptable internal consistency reliability for the CAAS (Savickas & Porfeli, 2012; Zewude et al., 2025a).

In the present study, the scale demonstrated strong reliability and convergent validity. Specifically, the indices for Concern (α = 0.926, CR = 0.942, AVE = 0.732), Confidence (α = 0.903, CR = 0.925, AVE = 0.674), Control (α = 0.919, CR = 0.937, AVE = 0.713), and curiosity (α = 0.922, CR = 0.939, AVE = 0.719), all exceeded the recommended thresholds of 0.70 for Cronbach’s alpha and composite reliability, and 0.50 for average variance extracted. Discriminant validity was also supported, as the HTMT values ranged from 0.274 to 0.663, and the correlations among constructs were lower than the square roots of their respective AVE values.

### 2.4. Statistical Data Analysis

To analyze the data, the study utilized two statistical software packages, including Statistical Package for the Social Sciences (SPSS) version 29, and Smart PLS 4.1.1.5 (Ringle et al., 2024). The selection of Partial Least Squares Structural Equation Modeling (PLS-SEM) is highly relevant in psychological research, particularly when dealing with complex models and latent constructs (Ringle et al., 2024). PLS-SEM is a statistical technique adept at modeling complex cause-effect relationships involving both observed variables and latent variables, the unobservable phenomena like attitudes, perceptions, and psychological capital that are central to psychology (Hair et al., 2022). A key advantage of PLS-SEM is its robust capability in handling complex models with numerous constructs and relationships, which is common when integrating multiple psychological theories and it is often favored in exploratory research aimed at theory development or when the theoretical foundation is not fully established, as it allows researchers to build and test theoretical models effectively (Hair et al., 2022). Crucially, it is less reliant on strict distributional assumptions, such as data normality, and is suitable for predictive modeling, making it a versatile tool for examining how psychological factors interrelate and influence outcomes like career adaptability (Hair et al., 2022).

### 2.5. Harman’s Single-Factor Test for Common Method Bias

Common-method biases are significant considerations in social science research, particularly when self-reported measures are used. These biases can arise from various factors, including the design of the instruments, response formats, participant instructions, and the context in which the assessment is conducted. To mitigate these issues, this study employed several strategies following the guidelines of (Zewude et al., 2025a). First, experts in the relevant field evaluated the content validity of each item before administration, ensuring that they were both appropriate and relevant. Informed consent was obtained from all participants, and their identities were anonymized to protect confidentiality. Some items were reverse-scored to help counter potential response biases, and the predictor and criterion variables were sourced from diverse contexts to reduce the impact of a single perspective. Additionally, factor variance analyses were performed to address measurement errors. Following Harman’s single-factor test guidelines, the assessment for common-method bias showed a computed variance of 30.041%, which is significantly below the 50% threshold, indicating that there were no common-method biases affecting the study’s findings.

### 2.6. Procedures of the Studies

The Ethics Committee of the Institute of Teacher Education and Behavioral Sciences at Wollo University approved the research protocol. Next, the researchers approached the relevant university administrators to obtain permission to contact faculty members and students for data collection. Under the close supervision of the researchers, faculty members administered the questionnaire to students in regular classroom settings without any time limit. Informed consent was obtained from all participating students. To ensure anonymity, participants were instructed not to write any personal identifying information, such as their names.

## 3. Results

### 3.1. Measurement Model

This study employed Partial Least Squares Structural Equation Modeling (PLS-SEM) with Smart PLS 4.1.1.6 for both the measurement and structural models.

As stated in the instrument’s section and presented in Table 1 and Table 2, the measurement model estimation demonstrated adequate reliability and validity evidence for each first-order factor. In other words: (a) Cronbach’s alpha and composite reliability values exceeded the threshold (0.7) suggested in the literature, (b) the average variance extracted (AVE) is above the minimum criterion of 0.5, (c) each construct’s correlation with other constructs is less than the square root of its and (d) all heterotrait-monotrait ratio (HTMT) values were below 0.85, a conservative threshold (see Table 2). The second-order constructs also demonstrate acceptable reliability and validity: career adaptability (α = 0.728, CR = 0.826, AVE = 0.546), emotional intelligence (α = 0.79, CR = 0.852, AVE = 0.603), mindfulness (α = 0.822, CR = 0.874, AVE = 0.581), and psychological capital (α = 0.867, CR = 0.910, AVE = 0.716). The correlation coefficients among higher-order constructs are below the square root of each construct’s AVE, and all heterotrait-monotrait ratio (HTMT) values are below the threshold of 0.9, confirming the discriminant validity. The Variance inflation factor (VIF), as a collinearity measure, also suggests that collinearity is not an issue among the second-order constructs because the VIF values for constructs were below 5.

### 3.2. Structural Model

**Figure 1:**
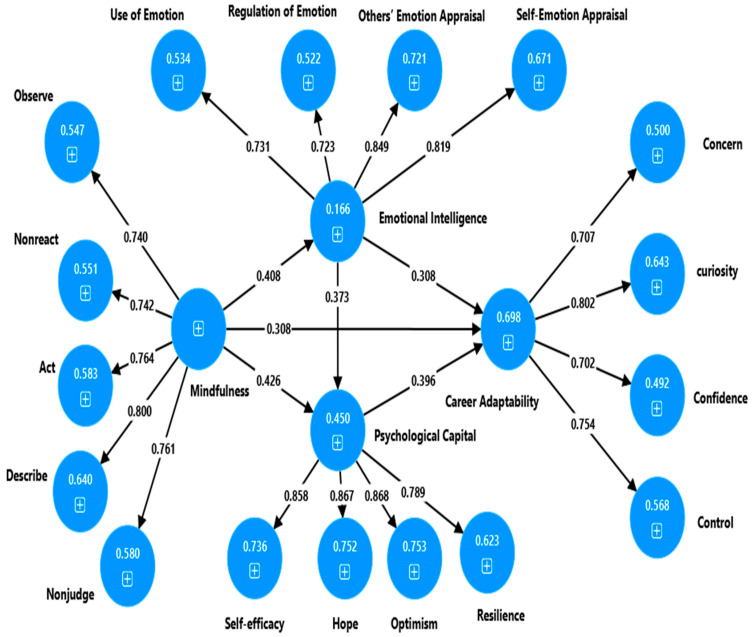
Mediation Pathways Linking Mindfulness to Career Adaptability through Emotional Intelligence and Psychological Capital.

The bootstrapping analysis, based on 5000 random resamples, provided empirical support for structural paths posited in the model. Specifically, as presented in Figure 1, the paths posited from mindfulness to emotional intelligence (β = 0.408, *p* < 0.001), psychological capital (β = 0.426, *p* < 0.001), and career adaptability (β = 0.308, *p* < 0.001) were positive and significant. Additionally, the direct positive effects of emotional intelligence on psychological capital (β = 0.373, *p* < 0.001) and career adaptability (β = 0.308, *p* < 0.001), as well as the influence of psychological capital on career adaptability (β = 0.396, *p* < 0.001), were statistically significant, confirming the hypothesized relationships.

### 3.3. Mediation Pathways

The bootstrap analysis revealed that both emotional intelligence (β = 0.126, BC 95% CI [0.093, 0.165], *p* < 0.001) and psychological capital (β = 0.168, BC 95% CI [0.127, 0.213], *p* < 0.001) partially mediated the path from mindfulness to career adaptability. In this study, a statistically significant, partially mediated effect of emotional intelligence on career adaptability, via psychological capital (β = 0.148, BC 95% CI = [0.108, 0.193], *p* < 0.001), was observed. The serial mediation path from mindfulness to career adaptability through emotional intelligence and psychological capital (β = 0.06, BC 95% CI = [0.043, 0.082], *p* = 0.001) was also statistically significant. Overall, the mediation analysis revealed that mindfulness demonstrated parallel and serial but partially mediated association with career adaptability. In the present structural model, both the direct and indirect influences of mindfulness on career adaptability are significant and positive, indicating that emotional intelligence and psychological capital serve as complementary mediators. The analysis examined the extent to which the predictors included in the model explain the outcome construct’s variance. The model was able to explain 69.8% of the variation in career adaptability (R^2^ = 0.698), which means that predictors were very important for understanding the outcome variable. Mindfulness and emotional intelligence explained 45% of the variance in psychological capital.

## 4. Discussion

The present study fully supported the proposed hypotheses. Specifically, the findings showed that: (a) mindfulness, emotional intelligence, and psychological capital each had direct effects on career adaptability; (b) mindfulness positively predicted both emotional intelligence and psychological capital; and (c) emotional intelligence and psychological capital partially mediated the relationship between mindfulness and career adaptability. In addition, the results supported a significant serial pathway in which mindfulness enhanced emotional intelligence, which in turn strengthened psychological capital and ultimately promoted career adaptability. Taken together, these findings suggest that mindfulness operates as a foundational psychological resource that directly and indirectly contributes to individuals’ capacity to manage career-related tasks, transitions, and uncertainties.

A key finding of this study is the significant direct effect of mindfulness on career adaptability. This result suggests that individuals with higher levels of mindfulness are better equipped to respond to career transitions, uncertainty, and developmental challenges. Mindfulness, commonly defined as present-moment awareness characterized by openness, attention, and nonjudgmental acceptance, has increasingly been recognized as an important personal resource in career development and workplace functioning (Candeias et al., 2021). Prior research has shown that mindfulness helps individuals cope with career uncertainty and change by promoting flexible, proactive, and adaptive responses (Zewude et al., 2025a; Zimmaro et al., 2016) In higher education settings, mindfulness has also been found to positively predict career adaptability among college students, including Korean undergraduates, emphasizing its importance in preparing young adults for dynamic career paths (Kim, 2022). Beyond career adaptability, mindfulness has been linked to career resilience and career success, suggesting that its benefits extend across multiple domains of vocational development (Zewude et al., 2025a; Zhang et al., 2025). More broadly, mindfulness contributes to positive workplace outcomes such as improved job performance, better interpersonal relationships, and enhanced well-being (Good et al., 2016). The present finding is therefore consistent with existing evidence suggesting that mindfulness supports adaptive career functioning.

One possible explanation for this association is that mindfulness reduces stress, strengthens emotional regulation, and improves decision-making. These psychological benefits may, in turn, facilitate the core dimensions of career adaptability. Specifically, mindfulness may support career concern by fostering future-oriented thinking, career control by encouraging self-directed decision-making, career curiosity by promoting openness to exploration, and career confidence by enhancing individuals’ belief in their ability to overcome obstacles and achieve career goals (Zewude et al., 2025a). From this perspective, mindfulness not only helps individuals remain attentive to present experiences but also enables them to respond constructively to future vocational demands.

The findings also indicated that emotional intelligence positively predicted career adaptability (Kim, 2022). This result is consistent with the career construction model (Savickas, 2005), which emphasizes the role of psychosocial resources in managing vocational development. It also aligns with prior empirical studies showing that emotional intelligence has a significant direct effect on career adaptability (Pong & Leung, 2023). Similarly, Mittal (2021) found that emotional intelligence, particularly the abilities to use and regulate emotions, plays an important role in career adaptability and job-search success. Extending this evidence, Pirsoul et al. (2023) reported that emotional intelligence was positively associated with career adaptability, career decision-making self-efficacy, entrepreneurial self-efficacy, salary, career commitment, career satisfaction, and entrepreneurial intentions, while being negatively associated with career decision-making difficulties and turnover intentions. Emotional intelligence has also been linked to broader workplace outcomes, including employee thriving and safety performance (Z. Wang et al., 2024). Collectively, these findings indicate that emotional intelligence enhances individuals’ capacity to understand, regulate, and use emotions effectively, thereby improving their ability to adapt to career tasks, transitions, and challenges.

Psychological capital also emerged as a significant positive predictor of career adaptability. This finding is consistent with previous research identifying psychological capital as an important personal resource for vocational adjustment (Ngoma & Dithan Ntale, 2016). For example, Huang et al. (2023), found that psychological capital had stronger predictive power for career adaptability than job satisfaction and life satisfaction. Likewise, Coetzee and Harry (2014) reported that psychological capital had direct effects on key aspects of career adaptedness, especially career resilience and career satisfaction. These results suggest that individuals with higher levels of hope, self-efficacy, resilience, and optimism are better prepared to cope with career-related demands and transitions (Coetzee et al., 2023). In this sense, psychological capital appears to function as a crucial motivational and adaptive resource that enhances effective career self-management.

With regard to the mediation model, the findings revealed that psychological capital partially mediated the relationship between mindfulness and career adaptability. This result highlights the role of mindfulness in fostering psychological capital, which subsequently contributes to stronger career adaptability (Huang et al., 2023). The finding is conceptually consistent with prior research showing that mindfulness helps build positive psychological resources, including self-efficacy, optimism, and resilience (Li et al., 2023; Zhao et al., 2022). Previous studies have also documented direct associations between psychological capital and career adaptability, as well as between core dimensions of psychological capital—such as hope, optimism, and resilience—and adaptive career outcomes (Buyukgoze-Kavas, 2016; Coetzee & Harry, 2014; Savickas & Porfeli, 2012). In addition, evidence supports a positive link between mindfulness and psychological capital (Li et al., 2023; Zhao et al., 2022). Therefore, the observed mediating effect is theoretically and empirically plausible. Students with stronger psychological capital may be more confident, resilient, and optimistic when facing career uncertainty, which in turn enables them to adapt more effectively. Psychological capital may thus serve as an important mechanism through which mindfulness is translated into adaptive career behavior.

The present study also found that emotional intelligence partially mediated the relationship between mindfulness and career adaptability. In exploring the psychological mechanisms underlying this association, emotional intelligence was considered a particularly salient variable because it reflects individuals’ capacity to perceive, understand, regulate, and use emotions in ways that facilitate adaptive functioning. The results are consistent with evidence indicating that emotional intelligence is significantly associated with career adaptability (Parmentier et al., 2019; Schutte & Malouff, 2011; Y. Wang et al., 2022), and that positive emotional dispositions contribute to adaptive career behavior (Coetzee & Harry, 2014). In addition, previous research has shown that mindfulness positively predicts emotional intelligence (Jiménez-Picón et al., 2021; Zhao et al., 2022), likely because mindfulness enhances self-awareness, attentional control, and emotional regulation. Although earlier studies have not directly examined emotional intelligence as a mediator between mindfulness and career adaptability, the present finding is theoretically well grounded. Since mindfulness promotes emotional intelligence, and emotional intelligence in turn supports career adaptability, it is reasonable to conclude that emotional intelligence serves as one explanatory pathway linking mindfulness to adaptive career outcomes.

Beyond the parallel mediation effects, this study provides evidence for a significant serial mediation pathway connecting mindfulness, emotional intelligence, psychological capital, and career adaptability. Specifically, mindfulness appears to act as a foundational resource that enhances emotional intelligence; emotional intelligence then contributes to the development of psychological capital; and psychological capital, in turn, strengthens career adaptability. This finding is theoretically meaningful because it suggests that adaptive career development may unfold through a chain of interrelated psychological resources. Individuals with greater mindfulness may be more capable of recognizing and regulating their emotions, which strengthens emotional intelligence. Higher emotional intelligence may then facilitate the development of psychological capital by helping individuals maintain hope, confidence, resilience, and optimism in the face of stress and uncertainty. These positive psychological resources, in turn, support effective adaptation to career transitions and challenges. This interpretation is consistent with prior literature showing that mindfulness improves emotional regulation (Keng et al., 2011), emotional intelligence is positively associated with psychological capital (Zewude et al., 2024b), and psychological capital supports adjustment to career transitions (B. C. Luthans et al., 2014). 

Overall, the present findings make an important contribution to the literature on career adaptability by clarifying both the direct and indirect roles of mindfulness. The study suggests that mindfulness is not only a personal trait associated with adaptive career functioning, but also a foundational resource that facilitates the development of other psychosocial capacities, especially emotional intelligence and psychological capital. These findings extend current understanding of how internal psychological resources jointly shape career adaptability and provide a more integrated account of the mechanisms through which mindfulness contributes to vocational development.

From a practical perspective, the findings underscore the importance of incorporating mindfulness, emotional intelligence, and psychological capital into educational, counseling, and career development interventions. Because these resources appear to operate both independently and sequentially in promoting career adaptability, interventions targeting them may be especially effective in helping students and young adults prepare for increasingly complex and uncertain career environments. Educators, counselors, and career practitioners may therefore benefit from designing programs that cultivate mindful awareness, emotional regulation, resilience, optimism, and self-efficacy (Zewude et al., 2024b). Strengthening these psychosocial resources may enhance not only career adaptability but also broader academic, personal, and occupational functioning.

## 5. Implications

Prior literature has established career adaptability as an indispensable personal resource that enables young people to respond effectively to changing conditions and associated challenges. It enhances their ability to utilize environmental resources, improve career prospects, and align themselves with available opportunities, thereby contributing to employability (Rossier et al., 2017). In today’s dynamic world of work, strengthening young people’s career adaptability is therefore essential. Research further shows that individuals with higher career adaptability are better able to manage unexpected changes in employment circumstances and recover more effectively from setbacks (Rudolph et al., 2017). Interventions that enhance career adaptability can thus strengthen individuals’ capacity to navigate career transitions and uncertainty, ultimately supporting long-term career success and growth.

The present study extends this literature by demonstrating both direct and indirect links between mindfulness and career adaptability, with emotional intelligence (EI) and psychological capital (PsyCap) serving as key mediating mechanisms. These findings contribute to career development theory by identifying psychological resources through which mindfulness may foster adaptability. In particular, they suggest that mindfulness is not only beneficial in itself, but also operates by strengthening individuals’ socio-emotional competence and positive psychological capacities, which are critical for adapting to career-related challenges (Zewude et al., 2025a). Because the current sample comprises university students at a pivotal stage of career identity formation and transition into the labor market, these findings carry important and actionable implications for higher education, student support services, and career counseling. The identified pathway from mindfulness to career adaptability—mediated by EI and PsyCap—suggests that interventions combining mindfulness training with the targeted development of socio-emotional competencies and positive psychological resources may meaningfully enhance students’ ability to manage career transitions (Hirschi et al., 2015; Hirschi, 2009; Howes & Goodman-Delahunty, 2015; Ng & Feldman, 2007; F. Luthans et al., 2007; B. C. Luthans et al., 2014).

More specifically, integrated interventions such as mindfulness-based workshops paired with emotional intelligence coaching and resilience or self-efficacy development modules may be especially effective. Such approaches are consistent with evidence showing that socio-emotional learning improves career decision-making and adaptive coping among emerging adults (Ng & Feldman, 2007; Lent & Brown, 2013). Compared with single-component interventions, these multi-component programs may produce greater and more enduring improvements in career adaptability, job-search self-efficacy, and early career outcomes.

Accordingly, universities should consider embedding such interventions across multiple student support contexts, including career centers, counseling services, first-year transition courses, career development seminars, and co-curricular programming. Educators and career counselors are particularly well positioned to implement mindfulness-based and psychologically informed programs that cultivate mindfulness, EI, and PsyCap together. Given the growing unpredictability of contemporary labor markets, fostering these resources may help students proactively navigate career transitions with greater resilience, confidence, and adaptability.

In practical terms, career education should move beyond a narrow focus on occupational information and job-search techniques to incorporate strategies that strengthen the psychological resources underpinning adaptive career behavior. Mindfulness, EI, and PsyCap are all developable within educational settings and may serve as foundational capacities for long-term career adaptability. Integrating these elements into career development programming may therefore offer a practical and scalable way to better prepare students for the demands of increasingly dynamic work environments.

Finally, we recommend that higher education institutions pilot and rigorously evaluate multi-component interventions that explicitly target mindfulness, emotional intelligence, and psychological capital. Future program evaluation should incorporate longitudinal follow-up and behavioral indicators to assess whether gains in these psychological resources translate into real-world outcomes, such as improved career decision-making, greater job-search engagement, smoother school-to-work transitions, and stronger early employment trajectories. In this way, the present findings not only advance the career development literature but also provide a basis for designing evidence-informed interventions that support students’ successful entry into contemporary labor markets.

## 6. Conclusions

In summary, this study provides robust evidence that mindfulness plays a central role in promoting career adaptability. Mindfulness demonstrated a significant direct effect on career adaptability and also influenced it indirectly through two key psychological resources—emotional intelligence and psychological capital. Specifically, emotional intelligence and psychological capital were shown to partially mediate the mindfulness–career adaptability relationship, indicating that mindfulness enhances adaptive career functioning by strengthening these internal capacities. Moreover, the findings further supported a serial mediation pathway in which mindfulness enhances emotional intelligence, which then fosters psychological capital, ultimately leading to improved career adaptability.

Theoretically, these results extend understanding of how mindfulness operates as a foundational personal resource within career development processes. Practically, they highlight the value of integrating mindfulness-based approaches with interventions designed to strengthen emotional competencies and psychological capital. Such integrated programs may be particularly effective for helping students and young adults manage career transitions, uncertainty, and developmental challenges in increasingly dynamic career environments.

## 7. Limitations and Future Research Directions

Several limitations in this study motivate specific future research actions. Because the study is cross-sectional, it provides only a snapshot and therefore cannot support causal interpretations; the direction among mindfulness, career adaptability, EI, and PsyCap remains uncertain. To address this, future research should adopt longitudinal, multi-wave designs (across semesters/academic years) to establish temporal ordering and test whether mindfulness predicts later increases in PsyCap/EI, which then enhance career adaptability (Zewude et al., 2025a). Relatedly, experimental and quasi-experimental designs, including randomized controlled trials of mindfulness-based interventions, should be used to test whether increasing mindfulness produces downstream changes in PsyCap/EI and ultimately career adaptability (Zewude et al., 2025a).

In addition, the reliance on self-report measures creates risks of reporting bias and common-method variance (CMV) because all constructs were assessed via the same questionnaire in one administration. Although Harman’s single-factor test and marker-variable sensitivity analyses suggested CMV is unlikely to dominate, these checks cannot fully rule out shared method effects. Future studies should therefore reduce common-method concerns using multi-source/multi-method strategies (e.g., time-lagged measurement, peer/teacher ratings, institutional records, and behavioral indicators of career exploration) to confirm whether observed relationships persist beyond self-report (Coetzee et al., 2023; Tjimuku et al., 2025; Zewude et al., 2025a).

The study also faces measurement relevance limitations: questionnaire items may not fully capture contextually grounded functioning of mindfulness, EI, and PsyCap during concrete career events (e.g., internships, job search, and school-to-work transition decisions). Future research should improve measurement precision by combining questionnaire measures with context-specific behavioral or event-based assessments, so that psychological resources can be linked more directly to real career behaviors and workplace outcomes (Coetzee et al., 2023; Tjimuku et al., 2025).

Moreover, the cultural and contextual specificity of the sample constrains generalizability because career adaptability and psychological resources may be shaped by collectivistic norms, relational expectations, and culturally meaningful interpretations of career development. Future research should expand into cross-cultural studies that test measurement invariance and replicate the model across diverse cultural and labor-market contexts to determine whether pathways are universal or culture-contingent (Coetzee & Harry, 2014; Coetzee et al., 2023).

The absence of some important covariates and boundary conditions (e.g., demographics, prior mindfulness exposure, academic/work experience, and individual differences) may also influence pathway stability. Future studies should incorporate these variables and test moderation and moderated mediation (e.g., gender, academic discipline/year, work/internship exposure, prior training, trait-level differences such as anxiety and personality) to clarify for whom and under what conditions mindfulness more effectively develops PsyCap/EI and improves career adaptability (Adeeb et al., 2026; Candeias et al., 2021).

Finally, the model may be incomplete: the tested mediators (partial mediation via PsyCap and EI) do not exhaust other mechanisms through which mindfulness could support career adaptability (e.g., emotion regulation, perceived stress, self-compassion, proactive career behaviors, self-regulated learning). Future research should therefore broaden the mediating framework by testing additional theoretically relevant pathways to clarify “how” mindfulness translates into adaptability through affective, motivational, cognitive, and social-interpersonal routes (Baluku et al., 2021; Coetzee & Harry, 2014; Tjimuku et al., 2025; Zewude et al., 2025a). In addition, integrating meaning-centered/existential variables (e.g., meaning in life) may deepen the explanation for the “why” behind career adaptability and potentially strengthen the mindfulness → PsyCap/EI → adaptability pathway (Adeeb et al., 2026).

## Figures and Tables

**Table 1:** Reliability and Validity Evidence of First-order Factors.

No.	Variables	CA	CR	1	2	3	4	5	6	7	8	9	10	11	12	13	14	15	16	17
1	Act	0.82	0.89	**0.73**																
2	Concern	0.93	0.94	0.55	**0.73**															
3	Confidence	0.90	0.93	0.58	0.61	**0.67**														
4	Control	0.92	0.94	0.23	0.27	0.33	**0.71**													
5	Describe	0.87	0.91	0.48	0.18	0.30	0.55	**0.66**												
6	Hope	0.89	0.93	0.29	0.29	0.32	0.59	0.61	**0.82**											
7	Nonjudge	0.88	0.93	0.49	0.22	0.30	0.62	0.79	0.45	**0.81**										
8	Nonreact	0.83	0.89	0.69	0.68	0.48	0.31	0.46	0.30	0.42	**0.66**									
9	Observe	0.82	0.89	0.79	0.52	0.77	0.29	0.44	0.34	0.44	0.68	**0.73**								
10	Optimism	0.87	0.92	0.35	0.30	0.31	0.69	0.66	0.77	0.58	0.36	0.34	**0.79**							
11	Others’ emotion appraisal	0.87	0.91	0.27	0.35	0.32	0.56	0.31	0.62	0.31	0.23	0.34	0.48	**0.73**						
12	Regulation of emotion	0.83	0.89	0.21	0.18	0.20	0.43	0.36	0.25	0.34	0.18	0.23	0.27	0.45	**0.66**					
13	Resilience	0.83	0.90	0.43	0.46	0.70	0.61	0.50	0.69	0.44	0.39	0.56	0.69	0.55	0.40	**0.74**				
14	Self-emotion appraisal	0.85	0.90	0.26	0.35	0.29	0.70	0.40	0.59	0.43	0.28	0.27	0.66	0.83	0.43	0.56	**0.69**			
15	Self-efficacy	0.93	0.96	0.25	0.24	0.27	0.53	0.60	0.72	0.43	0.27	0.25	0.74	0.47	0.22	0.63	0.60	**0.88**		
16	Use of emotion	0.82	0.88	0.34	0.32	0.29	0.26	0.18	0.13	0.23	0.18	0.31	0.16	0.50	0.79	0.33	0.42	0.11	**0.65**	
17	Curiosity	0.92	0.94	0.39	0.38	0.35	0.66	0.57	0.80	0.45	0.30	0.35	0.74	0.70	0.31	0.62	0.77	0.69	0.42	**0.72**

Notes: The diagonal values (in bold) represent the average variance extracted, while the off-diagonal elements represent the heterotrait-montrait ratio (HTMT).

**Table 2:** Fornell-Larcker criterion.

No.	Variables	1	2	3	4	5	6	7	8	9	10	11	12	13	14	15	16	17
1	Act	**0.86**																
2	Concern	0.48	**0.86**															
3	Confidence	0.50	0.56	**0.82**														
4	Control	0.20	0.25	0.30	**0.84**													
5	Describe	0.41	0.16	0.27	0.50	**0.82**												
6	Hope	0.25	0.26	0.29	0.53	0.53	**0.90**											
7	Nonjudge	0.41	0.20	0.26	0.56	0.69	0.40	**0.90**										
8	Nonreact	0.57	0.60	0.42	0.27	0.39	0.26	0.36	**0.81**									
9	Observe	0.64	0.45	0.66	0.25	0.37	0.28	0.38	0.56	**0.86**								
10	Optimism	0.30	0.27	0.27	0.62	0.58	0.68	0.50	0.30	0.29	**0.89**							
11	Others’ emotion appraisal	0.23	0.31	0.29	0.50	0.27	0.54	0.27	0.20	0.29	0.42	**0.85**						
12	Regulation of emotion	0.18	0.16	0.18	0.37	0.31	0.21	0.29	0.15	0.19	0.23	0.39	**0.81**					
13	Resilience	0.35	0.40	0.61	0.53	0.43	0.59	0.38	0.32	0.46	0.58	0.47	0.33	**0.86**				
14	Self-emotion appraisal	0.21	0.31	0.26	0.63	0.34	0.51	0.37	0.23	0.23	0.56	0.72	0.37	0.47	**0.83**			
15	Self-efficacy	0.21	0.22	0.25	0.49	0.54	0.66	0.39	0.23	0.22	0.67	0.43	0.19	0.55	0.53	**0.94**		
16	Use of emotion	0.27	0.28	0.25	0.23	0.16	0.11	0.19	0.15	0.25	0.14	0.43	0.65	0.27	0.35	0.10	**0.80**	
17	Curiosity	0.34	0.36	0.32	0.61	0.51	0.73	0.41	0.26	0.30	0.66	0.63	0.28	0.55	0.68	0.65	0.36	**0.85**

Notes: The diagonal values (in bold) represent the square root of the average variance extracted, while the off-diagonal elements are the correlations between variables.

## Data Availability

The corresponding authors hold the data sets generated and analyzed during the study and are willing to share them upon request.
